# Comparison of outcomes between minimally invasive percutaneous bunnell suture and traditional open modified Kessler technique for acute closed achilles tendon rupture: A single-center cohort study

**DOI:** 10.1371/journal.pone.0339026

**Published:** 2025-12-15

**Authors:** Zihang Zhao, Xiang Zhang, Xi Hou, Zihan Liu, Zhiyong Hou, Lianxin Song, Ruipeng Zhang

**Affiliations:** 1 Department of Orthopaedic Surgery, Hebei Medical University Third Hospital, Shijiazhuang, Hebei, China; 2 Hebei Medical University, Shijiazhuang, Hebei, China; 3 Engineering Research Center of Orthopedic Minimally Invasive Intelligent Equipment, Ministry of Education, Shijiazhuang, China; 4 Key Laboratory of Biomechanics of Hebei Province, Shijiazhuang, Hebei, China; 5 NHC Key Laboratory of Intelligent Orthopaedic Equipment, Shijiazhuang, Hebei, China; 6 Key Laboratory of Precise Assessment, Diagnosis, and Treatment of Soft Tissue Injury of Hebei Province, Shijiazhuang, Hebei, China; 7 Department of Orthopaedic Surgery, Hebei Medical University Third Hospital, Shijiazhuang, Hebei, China; Chongqing University Three Gorges Hospital, CHINA

## Abstract

Percutaneous Bunnell repair and open modified Kessler repair remain debated options for acute Achilles tendon rupture (AATR). We retrospectively compared a minimally invasive percutaneous Bunnell technique (Group A) with an open modified Kessler repair (Group B) within a standardized early functional rehabilitation (EFR) protocol at a single center. Fifty-five adults with closed AATR treated between January 2021 and December 2022 were analyzed (Group A, n = 25; Group B, n = 30). Between-group comparisons used Welch t tests for continuous variables and χ² or Fisher exact tests for categorical variables; American Orthopaedic Foot & Ankle Society (AOFAS) and Achilles Tendon Total Rupture Score (ATRS) were assessed at 12 and 24 weeks, with Holm adjustment applied within each scale. Compared with Group B, Group A had shorter operative time (56.6 ± 15.1 vs 68.2 ± 23.2 minutes; mean difference −11.6; 95% CI −22.05 to −1.15; P = 0.030), less intraoperative blood loss (28.4 ± 8.4 vs 74.7 ± 19.4 mL; −46.3; 95% CI −54.22 to −38.38; P < 0.001), and a shorter length of stay (4.8 ± 2.0 vs 6.6 ± 2.9 days; −1.8; 95% CI −3.13 to −0.47; P = 0.009). At 12 weeks, AOFAS favored Group A (+1.36; 95% CI + 0.19 to +2.53; P = 0.047) and ATRS also favored Group A (+1.73; 95% CI + 0.48 to +2.98; P = 0.008). At 24 weeks, AOFAS was not different (+0.51; 95% CI −0.49 to +1.51; P = 0.312), whereas ATRS remained higher in Group A (+2.17; 95% CI + 0.88 to +3.46; P = 0.003). Overall complications were 12.0% vs 26.7% (risk difference −14.7%; 95% CI −34.10 to +7.20; P = 0.310); no sural nerve injuries occurred. Within an EFR framework, percutaneous Bunnell repair provided superior perioperative efficiency with comparable safety and modest, sub-MCID functional advantages, supporting confirmation in larger prospective studies.

## Introduction

Recent registry data show a sustained increase in the incidence of acute Achilles tendon rupture (AATR). Between 2002 and 2021, the incidence rose from approximately 28.8 to 41.7 per 100,000 person-years, with men accounting for nearly 80% of cases [1]. Many ruptures occur during abrupt, forceful athletic movements, disproportionately affecting male physical activity participants [2]. The injury can lead to significant pain, functional impairment, and prolonged disability if not managed appropriately [3–5].

Current treatment strategies include non-operative management and surgical repair. While operative intervention is often preferred for its lower re-rupture rates compared to non-operative approaches, the choice of surgical technique remains debated [6,7]. Open repair, such as the modified Kessler method, is widely used but carries a substantial wound complication burden, with reported infection rates ranging from 8% to 15% in contemporary series [8]. This significant morbidity underscores the clinical need for techniques that minimize soft-tissue disruption. Minimally invasive percutaneous techniques reduce soft-tissue dissection and may facilitate faster recovery. However, they carry a higher risk of sural nerve injury compared to open repair, as confirmed in recent comparative studies [6,9].

Direct comparative evidence between specific techniques remains limited. Systematic reviews have broadly contrasted minimally invasive and open categories, but few studies have directly compared percutaneous Bunnell-type repair with open core-suture techniques [10]. Recent clinical comparison has shown that the percutaneous Bunnell approach is associated with shorter operative times and fewer wound complications, with no clear disadvantage in re-rupture rates compared to open core-suture techniques [11]. Moreover, many prior comparisons did not standardize postoperative rehabilitation across groups, limiting interpretability in the context of modern accelerated protocols [12,13].

To address these evidence gaps, we conducted a single-center retrospective cohort study directly comparing percutaneous Bunnell and open modified Kessler repair for acute, closed Achilles tendon rupture. All patients followed a standardized early functional rehabilitation (EFR) protocol to minimize postoperative heterogeneity. This design enabled a focused evaluation of whether differences in surgical exposure and technique result in measurable advantages in perioperative efficiency, complication profile, and mid-term functional recovery.

## Methods

### Study design and ethical approval

This retrospective cohort study was conducted at the Trauma Emergency Center, Department of Orthopaedic Surgery, The Third Hospital of Hebei Medical University. The study protocol received ethical approval from the Institutional Review Board (IRB) of The Third Hospital of Hebei Medical University (Approval No. K2025-088-1). Given the retrospective design based on existing medical records, the IRB waived the requirement for individual informed consent. All procedures complied with the principles of the Declaration of Helsinki and followed the STROBE reporting guidelines.

### Participants

Consecutive adults with an acute, closed, unilateral midsubstance Achilles tendon rupture within one week of injury were screened. Diagnosis was confirmed by ultrasonography, with magnetic resonance imaging obtained when necessary. Inclusion criteria were: age 18–65 years, a complete midsubstance rupture approximately 3–6 cm proximal to the calcaneal insertion, and an ultrasound-measured tendon gap < 5 cm. Exclusion criteria included: rupture duration > 1 week; prior ipsilateral surgery or significant pathology; open, insertional, or chronic ruptures; pre-existing disability or limited ambulation that could affect rehabilitation; pregnancy; and inability to adhere to postoperative rehabilitation or scheduled follow-up.

### Treatment allocation and surgical techniques

All surgical procedures were performed by two senior orthopaedic trauma surgeons. The choice of surgical technique was determined by the surgeon’s clinical judgment and patient consultation rather than randomization. Decision factors included the tendon gap, tissue quality, local soft-tissue conditions, comorbidities, and patient preference. When both techniques were deemed equally suitable, a pragmatic alternation policy was applied to reduce selection bias.

**Fig 1:**
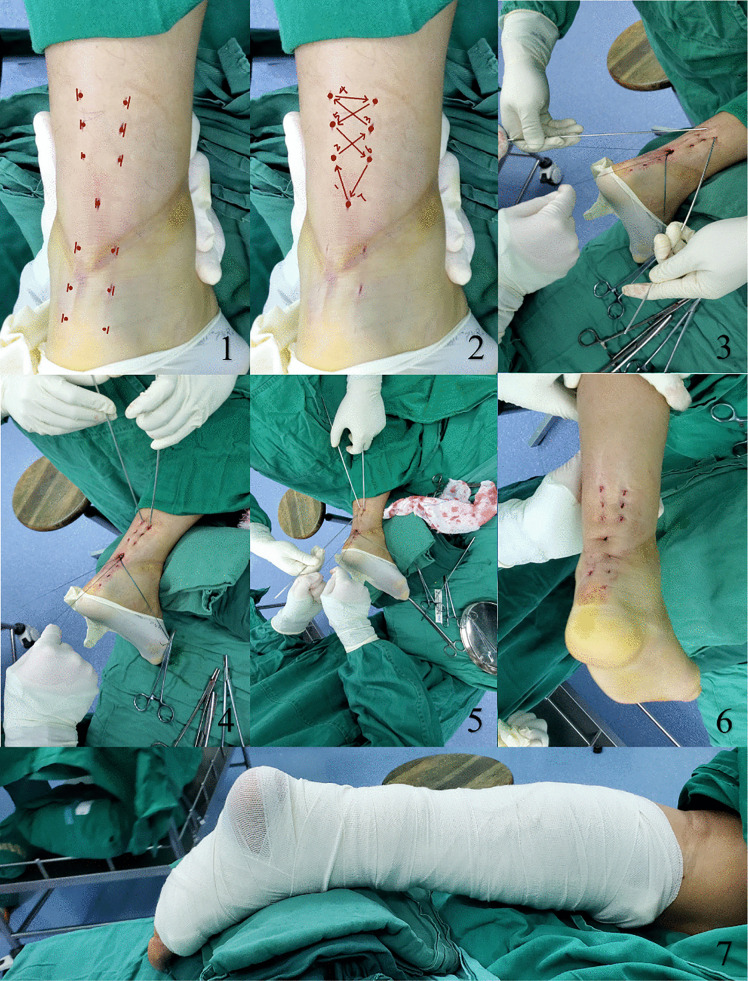
Steps of the minimally invasive percutaneous Bunnell repair.

Group A: Percutaneous Bunnell repair. Patients were positioned prone under spinal or epidural anesthesia, without a tourniquet. A 5-mm transverse incision was made over the rupture site to evacuate the hematoma. Additional small stab incisions were created proximally and distally to facilitate suture passage. Repairs were performed using a 2−0 non-absorbable suture on a curved 26-mm needle, employing a standardized Z-shaped weaving configuration with four passes per tendon end. The sutures were tensioned and tied with the ankle in plantar flexion (Fig 1).

① Minimally invasive percutaneous incision (transverse incision ~5 mm) at the rupture site; ② Schematic of the percutaneous Bunnell suture (2−0 non-absorbable suture on a curved 26-mm needle; standardized Z-shaped weaving with four passes per tendon end); ③–⑤ Reduction of a unilateral ruptured Achilles tendon using a prying technique; ⑥ Postoperative incision appearance; ⑦ Postoperative immobilization with a plaster cast in ankle plantar flexion position.

Group B: Open modified Kessler repair. Patients were positioned prone under spinal or epidural anesthesia with a thigh tourniquet. A 5–8-cm longitudinal incision was made along the posteromedial (or posterolateral) aspect of the leg, with meticulous care to avoid sural nerve injury. The tendon was repaired with two core sutures of 2−0 non-absorbable material using the modified Kessler technique and reinforced with epitendinous sutures. The paratenon was repaired whenever possible, and the wound was closed in layers.

### EFR protocol

All patients adhered to a uniform, standardized EFR protocol, which was administered by licensed physiotherapists specifically trained in its application. Adherence to the protocol was monitored through weekly outpatient visits and by reviewing patient-maintained exercise diaries. The rehabilitation regimen was designed to promote early mobilization and weight-bearing, in accordance with evidence suggesting these approaches can safely expedite functional recovery [14,15].

The staged protocol was structured as follows:

Weeks 0–2: Immobilization in a walking boot with heel wedges; non–weight-bearing with crutches; active toe flexion and isometric contractions of the plantar flexors.
Weeks 2–6: Gradual removal of heel wedges; progressive weight-bearing; initiation of controlled active ankle range-of-motion exercises and bilateral heel raises.
Weeks 6–8: Full weight-bearing in the boot with the ankle in a neutral position; advancement of strengthening, balance, and proprioception training.
Weeks 8–12: Transition to supportive footwear; continued strength and proprioceptive exercises; introduction of light-impact or sport-specific activities as tolerated.

Progression through each phase was contingent upon the absence of significant pain, swelling, or wound complications.

### Outcome measures

Data were retrospectively extracted from medical records by research staff blinded to the study hypotheses. The primary outcome was tendon re-rupture, monitored for 52 weeks. Secondary outcomes included postoperative complications, namely superficial and deep wound infections, symptomatic DVT confirmed by Doppler ultrasound, and sural nerve injury. Perioperative parameters were also assessed, including operative time, estimated intraoperative blood loss, and length of hospital stay. Functional recovery was evaluated using the American Orthopaedic Foot & Ankle Society (AOFAS) Ankle-Hindfoot Score and the Achilles Tendon Total Rupture Score (ATRS), with both patient-reported outcome measures collected at 12 and 24 weeks postoperatively.

### Study size

This retrospective cohort included all eligible patients treated during the study period. A formal a priori sample-size calculation was not performed because of the retrospective design. The study was conceived to provide exploratory estimates of variability and effect sizes to inform future prospective sample-size planning.

### Statistical analysis

Analyses were conducted using IBM SPSS Statistics for Windows, Version 27.0. Between-group comparisons of continuous variables at single time points (e.g., perioperative outcomes, baseline age) used Welch’s t-tests. Categorical variables were compared using χ² or Fisher’s exact tests, as appropriate. For functional scores at 12 and 24 weeks, between-group differences are reported as mean differences with 95% confidence intervals (Welch method). Holm’s procedure was applied within each scale to adjust P values across the two prespecified time points (12 and 24 weeks). To examine recovery trajectories, we computed within-patient change (Δ, 24–12 weeks) for each scale and compared Δ between groups using Welch’s t-test. For proportions, Wilson CIs are presented; for absolute risk differences, Newcombe–Wilson (method 10) CIs are reported. Standardized mean differences (SMDs) are provided for baseline characteristics (continuous variables via pooled SD; binary variables via the pooled-proportion form). Two-sided P < 0.05 was considered statistically significant.

## Results

### Patient enrollment and follow-up

**Fig 2:**
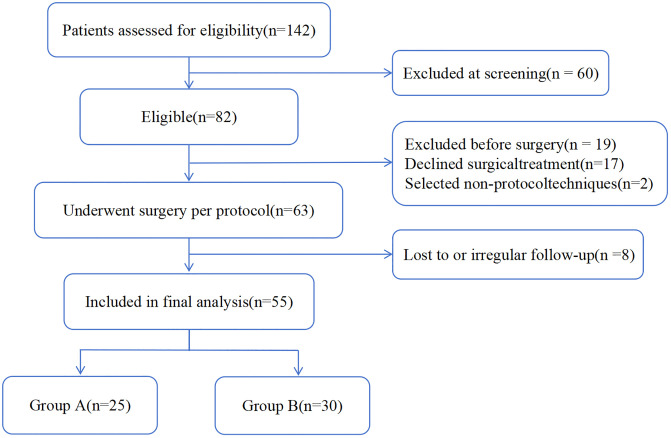
Flow diagram of patient screening, eligibility, and inclusion.

From January 2021 to December 2022, 142 patients were screened. Of these, 60 were excluded at screening, leaving 82 deemed eligible. During the preoperative phase, an additional 19 patients were excluded (17 declined surgical treatment, 2 opted for non-protocol techniques), resulting in 63 patients undergoing surgery. Over follow-up, 8 patients were lost to regular assessments or had irregular follow-up, leaving 55 patients with complete data on key functional and safety endpoints. All 55 patients completed the 12- and 24-week functional assessments and the 52-week safety follow-up and were included in the final analysis (25 in Group A, 30 in Group B) (Fig 2).

A total of 142 patients were assessed for eligibility. After exclusions, 63 underwent surgery per protocol, and 55 were included in the final analysis (Group A, n = 25; Group B, n = 30).

### Baseline demographics and clinical characteristics

Baseline characteristics are summarized in Table 1. The two groups were generally well balanced, with no statistically significant differences in demographic or clinical variables. Mean age was 39.8 ± 8.4 years in Group A and 41.4 ± 10.6 years in Group B. The cohort was predominantly male (100.0% in Group A vs 90.0% in Group B). Time from injury to surgery, side of injury, BMI, smoking status, and diabetes prevalence were comparable. Standardized mean differences indicated negligible to moderate imbalance overall.

**Table 1:** Baseline characteristics of the study population.

Variable	Group A (n = 25)	Group B (n = 30)	SMD	P value
Age, years (mean ± SD)	39.8 ± 8.4	41.4 ± 10.6	−0.17	0.535
BMI, kg/m² (mean ± SD)	26.3 ± 3.2	27.1 ± 3.6	−0.23	0.387
Time from injury to surgery, days (mean ± SD)	5.1 ± 2.3	5.6 ± 2.5	−0.21	0.444
Male, n/N (%)	25/25 (100.0%)	27/30 (90.0%)	+0.44	0.242
Current smoker, n/N (%)	4/25 (16.0%)	5/30 (16.7%)	−0.02	1.000
Diabetes, n/N (%)	1/25 (4.0%)	2/30 (6.7%)	−0.12	1.000
Right side injured, n/N (%)	16/25 (64.0%)	15/30 (50.0%)	−0.29	0.297

Data are presented as mean ± SD or n/N (%), as appropriate. P values are from Welch’s t-tests for continuous variables and two-sided Fisher’s exact tests for categorical variables. SMD denotes standardized mean difference (assessing balance between groups; sign reflects direction with Group A as reference).

BMI, body mass index; SD, standard deviation; SMD, standardized mean difference.

### Perioperative outcomes

As shown in Table 2, Group A had significantly shorter operative time (56.6 ± 15.1 min vs 68.2 ± 23.2 min; mean difference = −11.6; 95% CI −22.05 to −1.15; P = 0.030) and lower intraoperative blood loss (28.4 ± 8.4 mL vs 74.7 ± 19.4 mL; mean difference = −46.3; 95% CI −54.22 to −38.38; P < 0.001). Hospital stay was also shorter in Group A by 1.8 days (4.8 ± 2.0 vs 6.6 ± 2.9 days; 95% CI −3.13 to −0.47; P = 0.009).

**Table 2:** Perioperative outcomes.

Outcome	Group A (n = 25)	Group B (n = 30)	Between-group difference (95% CI)	P value
Operative time, min	56.6 ± 15.1	68.2 ± 23.2	−11.60 (−22.05 to −1.15)	0.030^*^
Blood loss, mL	28.4 ± 8.4	74.7 ± 19.4	−46.30 (−54.22 to −38.38)	< 0.001^*^
Length of stay, days	4.8 ± 2.0	6.6 ± 2.9	−1.80 (−3.13 to −0.47)	0.009^*^

Values are mean ± SD. Between-group comparisons used Welch’s t-tests; 95% CIs use the Welch method. * P < 0.05 after Holm adjustment within table.

SD, standard deviation.

### Functional outcomes at 12 and 24 Weeks

Functional outcomes are presented in Table 3. At 12 weeks, the mean AOFAS score was 90.8 ± 1.9 in Group A vs 89.4 ± 2.4 in Group B (mean difference = +1.36; 95% CI + 0.19 to +2.53; P = 0.047). The mean ATRS score at 12 weeks differed by +1.73 (90.8 ± 1.7 vs 89.1 ± 2.9; 95% CI + 0.48 to +2.98; P = 0.008).

**Table 3:** Functional outcomes at 12 and 24 weeks.

Outcome	Group A (n = 25)	Group B (n = 30)	Between-group difference (95% CI)	P value
AOFAS 12 weeks	90.8 ± 1.9	89.4 ± 2.4	+1.36 (+0.19 to +2.53)	0.047^*^
AOFAS 24 weeks	93.5 ± 1.8	93.0 ± 1.9	+0.51 (−0.49 to +1.51)	0.312
ATRS 12 weeks	90.8 ± 1.7	89.1 ± 2.9	+1.73 (+0.48 to +2.98)	0.008^*^
ATRS 24 weeks	93.8 ± 2.4	91.6 ± 2.3	+2.17 (+0.88 to +3.46)	0.003^*^

Values are mean ± SD. Between-group comparisons used Welch’s t-tests; 95% CIs use the Welch method. * P < 0.05 after Holm adjustment within table.

AOFAS, American Orthopaedic Foot & Ankle Society score; ATRS, Achilles Tendon Total Rupture Score; SD, standard deviation.

At 24 weeks, the AOFAS difference was not significant (93.5 ± 1.8 vs 93.0 ± 1.9; + 0.51; 95% CI −0.49 to +1.51; P = 0.312), whereas ATRS remained higher in Group A (93.8 ± 2.4 vs 91.6 ± 2.3; + 2.17; 95% CI + 0.88 to +3.46; P = 0.003). Although statistically significant, these PROM differences were below commonly cited MCIDs.

Change Scores (Δ = 24–12 Weeks).

Within-patient changes are shown in Table 4. The AOFAS change favored Group B (mean difference −0.85; 95% CI −1.95 to +0.25; P = 0.126), and the ATRS change showed no clear difference (mean difference +0.44; 95% CI −0.85 to +1.73; P = 0.497), indicating broadly parallel recovery trajectories.

### Complication rates

Complication data are detailed in Table 5. The overall complication incidence was 12.0% (95% CI 4.20 to 30.00) in Group A and 26.7% (95% CI 14.20 to 44.40) in Group B, yielding a risk difference of −14.7% (95% CI −34.10 to +7.20; P = 0.310). One re-rupture occurred in Group B (3.3%; 95% CI 0.60 to 16.70); none in Group A (0.0%; 95% CI 0.00 to 13.30). No superficial infections were recorded in Group A, whereas three cases (10.0%; 95% CI 3.50 to 25.60) occurred in Group B (risk difference −10.0%; 95% CI −25.60 to +4.80; P = 0.242). Symptomatic DVT rates were similar (12.0% vs 13.3%; risk difference −1.3%; 95% CI −19.50 to +18.30; P = 1.000). No sural nerve injuries were observed in either group. None of the between-group differences reached statistical significance. All three DVT cases were confirmed by duplex ultrasound and treated with therapeutic anticoagulation, achieving full resolution without sequelae. The three superficial wound infections in Group B were managed with oral antibiotics and local wound care; none progressed to deep infection.

## Discussion

This study demonstrates that under a standardized EFR protocol, percutaneous Bunnell repair offers clear perioperative advantages over open modified Kessler repair, while yielding no significant differences in tendon re-rupture, complication rates, or mid-term patient-reported functional outcomes.

Group A conferred clear benefits in perioperative efficiency. The nearly 12-minute shorter operative time observed in this study is consistent with findings in recent reports which documented substantially reduced surgical durations in minimally invasive versus open repair [16]. This advantage is also supported by meta-analytic comparisons across open, percutaneous, and mini-open techniques [9]. The decrease in intraoperative blood loss mirrors the pattern observed in limited open repair series emphasizing reduced soft-tissue disturbance [17]. The significantly shorter hospital stay in Group A further underscores a tangible clinical benefit, likely reflecting lower postoperative wound burden, reduced pain, and faster early recovery. Observationally, learning-curve studies report that once proficiency is achieved, minimally invasive operative time stabilizes, which strengthens the argument that percutaneous approaches can sustain efficiency advantage [18]. It should be noted that in our Methods we used different tourniquet strategies (none in Group A vs thigh tourniquet in Group B), which could partially influence blood loss and surgical field dynamics; future trials should standardize or stratify for tourniquet use.

The incidence of tendon re-rupture was low and comparable between groups, with one failure occurring Group B and none in Group A. This result suggests that applying the percutaneous Bunnell technique under a structured rehabilitation regimen did not increase rupture risk. This finding aligns with contemporary studies and meta-analyses reporting similarly low failure rates for modern surgical techniques [19,20]. A trend toward fewer superficial wound complications was also observed with the minimally invasive approach, consistent with prior evidence [14,21].

Our 52-week follow-up period aligns with the established timeline of substantial tendon maturation. Molecular and histological studies indicate that the repair tissue begins adopting tendon-like characteristics by approximately 10 weeks, with the subsequent remodeling phase—characterized by extracellular matrix reorganization and collagen alignment—progressing toward greater stiffness and strength over the following months [22,23]. Clinically, this transformative period is reflected in the finding that most patients return to sport around 6 months post-injury [24]. Therefore, although complete biological remodeling is a prolonged process, our 52-week surveillance window effectively captures this pivotal window of mechanical consolidation and is thus well-suited for assessing medium-term structural and clinical outcomes.

Biomechanical studies provide a mechanistic basis for our clinical findings. Controlled experiments show that Bunnell and modified Kessler repairs yield similar ultimate strength and gap resistance, with the Bunnell technique often exhibiting superior performance in cyclic loading tests [25,26]. The evidence confirms that both techniques, when proficiently executed, provide sufficient mechanical integrity to support early rehabilitation, thereby offering a biomechanical rationale for their comparable clinical outcomes.

Functional outcomes, assessed via AOFAS and ATRS scores, improved significantly in both groups from 12 to 24 weeks, with high absolute levels. Although small differences at individual time points slightly favored the group A, those differences fell below the commonly accepted MCID threshold for ATRS, which has been estimated in the literature as about 8–10 points based on the UKSTAR trial [27,28]. More importantly, comparison of within-patient change scores between 12 and 24 weeks showed no divergence in recovery trajectories (Table 4). Our use of the same rehabilitation protocol in both groups helps isolate the impact of surgical technique itself, reducing confounding from variable postoperative regimens and thus increasing the interpretability and clinical relevance of our findings.

**Table 4:** Change scores (Δ = 24–12 weeks).

Outcome	Group A (n = 25)	Group B (n = 30)	Between-group difference (95% CI)	P value
AOFAS Δ	+2.7 ± 1.4	+3.6 ± 1.2	−0.85 (−1.95 to +0.25)	0.126
ATRS Δ	+3.0 ± 1.4	+2.6 ± 1.3	+0.44 (−0.85 to +1.73)	0.497

Values are mean ± SD. Between-group comparisons used Welch’s t-tests; 95% CIs use the Welch method.

Δ, change; SD, standard deviation; AOFAS, American Orthopaedic Foot & Ankle Society score; ATRS, Achilles Tendon Total Rupture Score.

**Table 5:** Complications and between-group risk differences.

Outcome	Group A (n = 25)	Group B (n = 30)	Risk difference (A − B) (95% CI)	P value
Any complication	3/25 (12.0%)	8/30 (26.7%)	−14.70 (−34.10 to +7.20)	0.310
Re-rupture	0/25 (0.0%)	1/30 (3.3%)	−3.30 (−16.70 to +10.30)	1.000
Superficial infection	0/25 (0.0%)	3/30 (10.0%)	−10.00 (−25.60 to +4.80)	0.242
DVT	3/25 (12.0%)	4/30 (13.3%)	−1.30 (−19.50 to +18.30)	1.000

Data are expressed as n/N (%). Comparisons between groups used Fisher’s exact test. Risk differences are shown with 95% CIs using the Newcombe–Wilson (method 10) approach.

CI, confidence interval; DVT, deep vein thrombosis.

This single-center retrospective study carries inherent selection bias, although we have detailed the allocation rationale and demonstrated baseline comparability. The sample size, while sufficient to detect clinically relevant differences in perioperative outcomes, provides limited power for robust analysis of rare complications and precludes meaningful subgroup analyses. Objective biomechanical measures (e.g., strength testing, gait analysis) and long-term follow-up beyond one year were not available. Differences in tourniquet strategy between groups may have affected blood-loss comparisons, as noted above. The single-center setting and involvement of two senior surgeons may also affect the generalizability of our findings.

This study provides a direct clinical comparison of the percutaneous Bunnell configuration against a common open technique, distinct from device-assisted minimally invasive systems, within a standardized rehabilitation pathway. The significant perioperative advantages—operational efficiency and a favorable wound complication trend—coupled with non-inferior safety and functional outcomes, position the minimally invasive Bunnell technique as a compelling surgical option. It may be particularly advantageous in clinical scenarios prioritizing rapid recovery, soft-tissue preservation, or in patients at elevated risk for wound complications.

## Conclusion

Within a standardized EFR framework, the minimally invasive percutaneous Bunnell repair for acute Achilles tendon rupture demonstrated favorable perioperative efficiency and comparable safety and functional recovery relative to the open modified Kessler technique. These findings support the percutaneous Bunnell approach as a practical and effective option in selected patients. Further prospective, randomized multicenter studies with larger cohorts and long-term follow-up are warranted to confirm these results and refine patient selection criteria.
